# Multi-Edge Gene Set Networks Reveal Novel Insights into Global Relationships between Biological Themes

**DOI:** 10.1371/journal.pone.0045211

**Published:** 2012-09-13

**Authors:** Jignesh R. Parikh, Yu Xia, Jarrod A. Marto

**Affiliations:** 1 Bioinformatics Program, Boston University, Boston, Massachusetts, United States of America; 2 Department of Chemistry, Boston University, Boston, Massachusetts, United States of America; 3 Department of Biomedical Engineering, Boston University, Boston, Massachusetts, United States of America; 4 Department of Cancer Biology and Blais Proteomics Center, Dana-Farber Cancer Institute, Boston, Massachusetts, United States of America; 5 Department of Biological Chemistry and Molecular Pharmacology, Harvard Medical School, Boston, Massachusetts, United States of America; Semmelweis University, Hungary

## Abstract

Curated gene sets from databases such as KEGG Pathway and Gene Ontology are often used to systematically organize lists of genes or proteins derived from high-throughput data. However, the information content inherent to some relationships between the interrogated gene sets, such as pathway crosstalk, is often underutilized. A gene set network, where nodes representing individual gene sets such as KEGG pathways are connected to indicate a functional dependency, is well suited to visualize and analyze global gene set relationships. Here we introduce a novel gene set network construction algorithm that integrates gene lists derived from high-throughput experiments with curated gene sets to construct co-enrichment gene set networks. Along with previously described co-membership and linkage algorithms, we apply the co-enrichment algorithm to eight gene set collections to construct integrated multi-evidence gene set networks with multiple edge types connecting gene sets. We demonstrate the utility of approach through examples of novel gene set networks such as the chromosome map co-differential expression gene set network. A total of twenty-four gene set networks are exposed via a web tool called MetaNet, where context-specific multi-edge gene set networks are constructed from enriched gene sets within user-defined gene lists. MetaNet is freely available at http://blaispathways.dfci.harvard.edu/metanet/.

## Introduction

Networks that connect biomolecules such as genes and proteins with each other have been increasingly used to understand global cellular systems. Edges in biological networks are either determined from high-throughput experiments such as those for identifying physical interactions between protein pairs and potential transcription regulatory interactions between transcription factors and genes or are constructed using informatics approaches as in the case of gene co-expression networks. Informatics approaches that integrate various edge types as independent sources of evidence have been used to construct functional linkage networks for the prediction of gene function and identification of functionally coherent modules [1]. While multiple evidences collectively increase confidence in prediction of functional relationships, each edge type lends itself to its own unique interpretation.

Networks can also be used to describe functional relationships between biological themes [2] as defined by curated gene sets from databases such as Gene Ontology (GO) [3] and the Kyoto Encyclopedia of Genes and Genomes (KEGG) Pathway database [4]. A Gene set network connects related gene sets as opposed to individual genes, thus providing the benefits in visualization and established graph-theoretic methods of single-biomolecule networks with the added reduction in complexity via increased granularity. Some relationships such as the hierarchically organized GO terms are explicitly described as a directed acyclic graph (DAG) where child term definitions are subsets of parent terms. While others such as pathway crosstalk can be derived from the overlap between pathway gene sets. There have been several efforts such as ConceptGen [5] and the Molecular Concepts Map (MCM) [6] to generate global gene set networks based on co-membership where a pair of gene sets is connected if there are a significant number of genes in common between the two gene sets. Leveraging existing single-biomolecule networks such as protein-protein interaction and gene co-expression networks, Dotan-Cohen *et al*. describe a gene set network algorithm for the construction of biological process linkage networks where two GO biological process terms are linked in a gene set network if there are a significant number of edges between the unique gene members of the two terms in the original single-biomolecule network [7]. Unlike the GO DAG, which is specific to gene ontology terms, the aforementioned co-membership and linkage approaches can be applied to any generalized gene set collection to construct gene set networks with edge types that lend themselves to their own unique interpretations.

Li *et al*. [8] apply the linkage approach using protein-protein interaction data to construct a global pathway crosstalk network with pathway gene sets collected from GO, HumanCyc [9], and BioCarta; the authors identify co-membership as a potential source of redundancy and remove any linkage-based edges from the gene set network where there are also a significant number of shared genes between the two connected pathways. However, co-membership may reveal novel and non-redundant gene set pairs such as those between drug signatures and pathways [5], [6] identified in the ConceptGen and MCM gene set networks. A comparison between gene set relationships as determined by linkage versus co-membership approaches would provide resolution to the redundancy problem while highlighting potentially unique gene set relationships. Despite the availability of independently constructed gene set networks and algorithms for constructing novel gene set networks, there does not exist to our knowledge a repository of different types of gene set networks of popular gene set collections that have been constructed using a consistent framework.

Here, we describe a novel algorithm for constructing co-enrichment gene set networks that integrate experimentally derived gene lists with literature-curated gene sets and demonstrate the utility of the approach through examples of novel insights gained as compared to co-membership and linkage gene set networks when applied to KEGG Pathway and Chromosome Map gene sets through integration with gene expression microarray data. We take advantage of disease related gene sets in the KEGG Pathway database to compare neurodegenerative diseases based on their transcriptional dependencies with pathways. The relationship between differential expression, pathways, and linear and three-dimensional genome organization is demonstrated via the Chromosome Map co-enrichment gene set network. Next, we describe Phosphorylation Substrates gene set networks where gene sets consisting of substrates of kinases and phosphatases are connected to identify functional relationships between the enzymes, which to our knowledge are novel. We find that co-enrichment of Phosphorylation Substrates gene sets based on integration with gene expression does not provide additional information as compared to co-membership and linkage gene set networks, which is expected considering the post-translational nature of the gene sets.

Several tools such as the ConceptGen [5], Enrichment Map [10], and ClueGO have been developed to construct custom co-membership gene set networks connecting gene sets that are over represented within a user-inputted gene list. Alongside construction algorithms, network visualization tools such as VisANT [11] and Cytoscape [12] have been developed to support gene set network visualization, which take a bottom-up approach by collapsing sets of gene or proteins into meta-nodes. Given that co-enrichment, co-membership, and linkage gene set networks provide complementary information, we have developed MetaNet, a feature-rich web resource for convenient access to gene set networks constructed using multiple algorithms applied to gene sets defined by each of KEGG Pathway, WikiPathways [13], the three GO namespaces, cytogenetic bands, host-virus interactions from VirusMINT [14], and phosphorylation substrates. MetaNet places overrepresented gene sets derived from user-defined gene lists in the context of multi-edge gene set networks of functionally related biological themes. MetaNet is freely available at http://blaispathways.dfci.harvard.edu/metanet/.

## Results

### A generalized framework for constructing diverse gene set networks

Here we describe the intuition behind gene set network construction. For details please see Methods.

**Figure 1:**
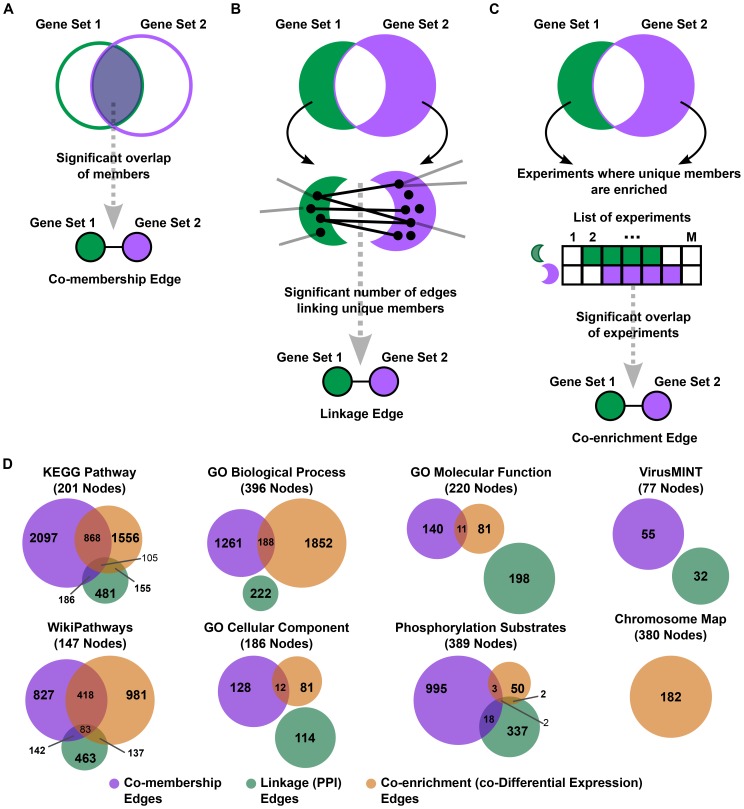
Generalized gene set network construction methods. A) Co-membership gene set networks connect gene sets if there is significant overlap in the gene set members. B) Linkage gene set networks connect a pair of gene sets if there are a significant number of edges between the unique components of the gene sets in a reference single-biomolecule network. C) Co-enrichment gene set networks connect gene sets if there are a significant number of experiments where the unique components of the gene sets are enriched together. D) The application of each of the three gene set network methods to 8 different gene set collections; the number of gene sets in each collection are noted in parentheses. The Venn diagrams describe the overlap in gene set pairs (edges) between two or all three gene set networks per collection.

Co-membership gene set networks connect a pair of gene sets if there is significant overlap between the corresponding members (Figure 1A). These gene set networks are constructed based on the curated gene set definitions alone and consequently do not depend on additional experimental data. Co-membership gene set networks, therefore, act as a baseline for comparison with other gene set networks to assess novel insight gained after integration of experimental data. It is important to note that the interpretation of co-membership varies per gene set collection. For example, co-membership of KEGG Pathway gene sets indicates pathway crosstalk while co-membership of GO molecular function gene sets may either indicate moonlighting functions or a shared ancestor in the GO DAG. Linkage gene set networks integrate curated gene sets with existing single-biomolecule networks by connecting gene set pairs if there are a significant number of links between the unique components of the two gene sets (Figure 1B); the meaning of an edge in the single-biomolecule network transfers to the gene set network. For example, a linkage gene set network constructed from a gene co-expression network would link co-expressed gene sets. In this study, we use physical interaction data to construct protein-protein interaction (PPI) gene set networks. Finally, co-enrichment gene set networks integrate curated gene sets with experimental gene lists and link two gene sets if the unique components of the two sets are consistently enriched together across many experimentally derived gene lists (Figure 1C). In this study, we integrate differentially expressed gene lists from gene expression microarray experiments to construct gene set networks describing co-differential expression of gene sets; the generalized co-enrichment approach can be applied to any collection of gene lists as long as the biological interpretation of each list is consistent. It is important to note that co-expression is distinct from co-differential expression in that co-expression measures concordant expression across many experiments while co-differential expression determines consistent and significant differential expression across many pairs of control-condition experiments. Between the aforementioned three generalized methods, a diverse set of gene set networks can be constructed. All three approaches share a common statistical framework for assessing significance of edges based on the Fisher's exact test (see Methods for details). The linkage and co-enrichment methods explicitly remove shared members to further contrast from the baseline co-membership relationships.

We apply the co-membership, linkage (PPI), and co-enrichment (differentially expressed gene lists) to popular collections of human gene sets, such as Gene Ontology (GO), KEGG Pathway, and WikiPathways, that are often used in gene set enrichment analyses (Figure 1D). For clarity, in the following sections we will refer to the linkage gene set networks from PPI data and the co-enrichment gene set networks from differentially expressed gene lists as PPI and co-DE (co-differential expression) gene set networks, respectively, to indicate the meaning of the edges. Based on the hierarchical ontology relationships, GO gene sets are subsets of others and consequently have perfect co-membership. In order to glean biological insight we eliminated GO gene sets that were too small or too large as well as those that were redundant within a lineage in order to avoid trivial GO DAG relationships (see Methods). Translocations, copy number variation, and abnormal karyotypes are often observed in cancers. Therefore, gene sets corresponding to chromosome loci as defined by cytogenetic bands are also analyzed. By definition, genes cannot be annotated as belonging to two distinct loci and consequently the Chromosome Map co-membership gene set network is null. Table 1 reports the most significant gene set pair in each of the three gene set networks per collection.

**Table 1:** Most significant edge in each gene set network.

	Co-membership		Co-PPI		Co-DE	
Gene set collection	Term 1	Term 2	Term 1	Term 2	Term 1	Term 2
**KEGG Pathway**	Oxidative phosphorylation	Parkinson's disease	Neuroactive ligand-receptor interaction	Long-term depression	Steroid biosynthesis	Terpenoid backbone biosynthesis
**WikiPathways**	Regulation of TLR signaling	TLR signaling	GPCRs, Class A Rhodopsin-like	Calcium Regulation in the Cardiac Cell	ErbB signaling	Estrogen signaling
**GO Biological** **Process**	MyD88-independent TLR signaling	TLR3 signaling	Exocytosis	Regulation of exocytosis	M/G1 Transition	Telomere maintenance
**GO Molecular** **Function**	Sequence-specific distal enhancer binding RNA polymerase II transcription factor activity	DNA binding, bending	Chemokine activity	Coreceptor activity	Integrin binding	Collagen binding
**GO Cellular** **Component**	Mitochondrial respiratory chain complex 1	Respiratory chain	Integrin complex	Lamellipodium membrane	Kinetochore	Spindle microtubule
**Chromosome Map**	None	None	None	None	chr8p11	chr8p12
**Phosphorylation** **Substrates**	PRKACG substrates	PRKX substrates	PRKACA substrates	SLC12A2 substrates	PIK3R1 substrates	MAP2K2 substrates
**VirusMINT**	HPV type 11 interactors	HPV type 16 interactors	HIV 1 interactors	HPV type 16 interactors	None	None

We also constructed gene set networks where gene sets were determined based on phosphorylation and physical interaction data (Table 1). A node in the Phosphorylation Substrates gene set networks corresponds to a collection of substrates of either a kinase or phosphatase as annotated in the literature curated databases KEGG Pathway, HPRD, and PhosphoSite Plus. We constructed VirusMINT gene set networks where each node representing a particular viral strain consisted of human proteins known to physically interact with proteins from the respective virus.

KEGG Pathway (1556 edges: 201 nodes), WikiPathways (981 edges: 147 nodes), and GO biological process (1852 edges: 396 nodes) co-DE gene set networks have the highest proportion of edges to nodes amongst the considered co-DE gene set networks suggesting that pairs of entire biological processes are more often transcriptionally controlled together than pairs of individual complexes or molecular functions (Figure 1D). The percentage of shared edges between co-membership and co-DE gene set networks is the highest for the pathway gene set collections (KEGG Pathway: 868/1556 = 56%, WikiPathways: 418/827 = 51%) indicating that the protein level crosstalk between pairs of pathways is reflected at the transcription level as well. Approximately half of the shared edges (403 edges) between the KEGG Pathway co-DE and co-membership gene set networks are incident to 15 cancer related pathway nodes. On the other hand, the two protein-interaction based co-DE gene set networks are negligibly sized or empty in the case of the Phosphorylation Substrates and VirusMINT gene set collections respectively. This is not surprising since post-translational regulation and host-virus interactions are not directly dependent of the transcriptional activity of the substrates. In four out of the eight collections, the co-membership gene set networks have the greatest number of edges relative to the other two gene set networks. The GO Molecular Function PPI gene set network and the Chromosome Map, WikiPathways, and GO Biological Process co-DE gene set networks have a greater number of edges than their respective co-membership gene set networks indicating that different type of gene set networks reveal varying amounts of information based on the gene set collection. Except the pathway collections, the gene set networks have minimal overlap in edges indicating that each of the three gene set networks provides complementary information regarding the gene set collections (Figure 1D). One explanation for any overlap between co-DE gene set network (or PPI gene set network) and co-membership gene set network edges is that the unique genes per gene set pair would be more related if there is a higher number (or percentage) of common genes removed and would thus cause the gene sets to be connected. To test this hypothesis we divided the edges per gene set network into those with higher or lower percentage of shared genes than the mean percentage of common genes for all possible overlapping gene set pairs (Table S1). We find, as expected, that a co-membership edge is more likely (85% of all edges) to occur if there is a higher than average overlap between gene sets. However, an edge is less likely, with a frequency of 19% and 21% in the co-DE and PPI gene set networks respectively, to occur if there is a higher than average overlap between gene sets; i.e. a high overlap is penalized since fewer genes remain in the individual gene sets. As a result, each of the aforementioned gene set networks (File S1) provide unique insights into global organization of aggregate biological entities.

In the following sections, we demonstrate the utility of gene set networks using the KEGG Pathway, Chromosome Map, and Phosphorylation Substrates gene set collections as examples. We first discuss co-DE as a novel approach for construction of global gene set networks.

### Comparing the KEGG pathway co-differential expression and co-membership gene set networks

The KEGG Pathway database contains literature-curated maps of metabolic, signaling, and immune response pathways as well as descriptions of cellular complexes such as the spliceosome and replication and repair machinery. The addition of disease related pathway maps provides for a common database for linking the aforementioned cellular processes with diseases. Furthermore co-membership between pathway gene sets directly lends itself to interpretation as pathway crosstalk, a well-studied phenomenon [15] that has been previously analyzed as a gene set network [8]. The KEGG Pathway co-DE gene set network aims to go beyond simple pathway membership and identify functional relationships between entire pathways, with the added advantage of providing hypotheses of co-regulation between biological processes and diseases.

**Figure 2:**
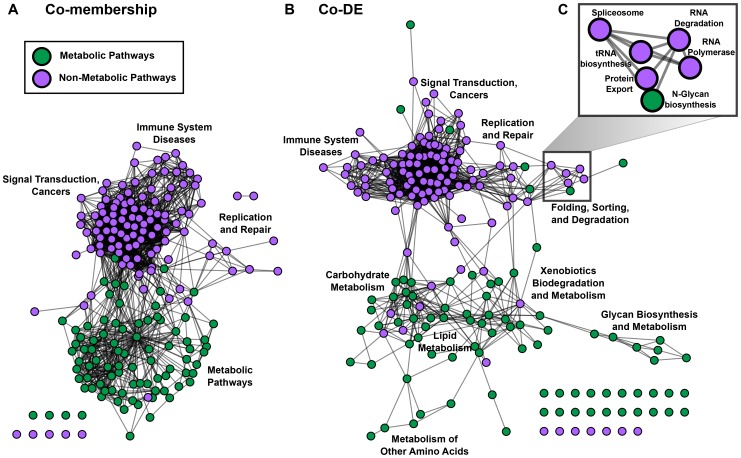
KEGG Pathway co-membership and co-differential expression gene set networks. In the KEGG Pathway gene set networks nodes represent KEGG Pathways; green nodes are metabolic pathways and purple nodes are non-metabolic pathways. A) The KEGG Pathway co-membership gene set network represents pathway crosstalk with an edge indicating a significant degree of crosstalk. B) The KEGG Pathway co-differential expression (co-DE) gene set network is constructed using the co-enrichment method applied to over five thousand differentially expressed gene lists derived from gene expression microarray data. C) A novel “Folding, Sorting, and Degradation” module is unique to the co-DE gene set network.

The KEGG Pathway gene set networks consist of 201 nodes each. After evaluating all 20100 possible pairs for pathways, we determined 2097 and 1556 edges in the co-membership and co-DE gene set networks respectively (Figure 1D and Figure 2). While sparse relative to the total number of possible edges, these two KEGG Pathway gene set networks are amongst the top three densest networks (see Methods for density calculation) in all the gene set networks in this study, emphasizing the fact that the KEGG Pathway database is well suited for studying relationships between fundamental cellular processes at the protein crosstalk and transcriptional level. In fact, the percentage of shared edges between the co-membership and co-DE gene set networks is the highest (56% overlap, p-value <1E-250 using Fisher's Exact Test) for KEGG Pathway gene sets suggesting that pathway crosstalk is reflected in gene expression above and beyond the shared biomolecules between pairs of cross-talking pathways. Metabolic pathways are clearly separated from other pathways in both gene set networks but the co-DE gene set network has a greater percentage of edges connecting a metabolic pathway node with a non-metabolic pathway node out of all edges incident to a metabolic pathway node (46% in the co-DE gene set network versus 32% in the co-membership gene set network). The proportional increase in cross-category edges suggests greater interplay at the transcriptional level between metabolic and non-metabolic pathways than at the protein pathway crosstalk level.

Despite the surprising similarities between the two gene set networks, considering that shared members are explicitly removed when constructing the co-DE gene set network, there are several unique relationships that are only revealed after integration of gene expression data. For example, the most significant co-DE edge is between the Steroid biosynthesis and Terpenoid backbone biosynthesis metabolic pathways (Table 1), an edge that is not found via co-membership considering there are zero enzymes that participate in both pathways; the edge is present in the KEGG Pathway PPI gene set network as well. Terpenoid backbone biosynthesis is a precursor to steroid biosynthesis and co-DE of the two processes suggests that they are coordinately controlled. Co-DE of disease related genes with pathways might also indicate coordinate regulation or functional dependence. The KEGG Pathway gene sets describing Alzhemier's disease, Huntington's disease, and Parkinson's disease have significant co-membership with each other; the three edges connecting the pairs of neurodegenerative diseases are amongst the top 5 most significant edges in the KEGG Pathway co-membership gene set network. There are several cancer pathways and signaling pathways that have significant co-membership with the three neurodegenerative diseases. However, it is well known that mitochondrial and other metabolic pathways are affected in each of the three diseases [16]–[19]. Except for Purine metabolism, Pyrimidine metabolism, Oxidative phosphorylation, and Citrate cycle (TCA), and the aggregate Metabolic pathways map, no specific metabolic pathways have significant co-membership with either disease. The KEGG Pathway co-DE gene set network reveals several metabolic pathways, i) TCA, ii) Fatty acid elongation in mitochondria, iii) Valine, leucine, and isoleucine degradation, iv) Pentose phosphate pathway, v) Glyoxylate and dicarboxylate metabolism, vi) Butanoate metabolism, vii) Tryptophan metabolism, and viii) Pyruvate metabolism, that are co-differentially expressed with the three diseases (Figure S1). The Fatty acid metabolism pathway is shared by Huntington's and Parkinson's disease but not by Alzheimer's disease. Glutathione metabolism is uniquely co-differentially expressed with Parkinson's disease (PD); gluatathione has been suggested as both a marker and therapy for PD [20]–[22]. Alzheimer's disease (AD), unlike Huntington's disease or PD, is also linked to several non-metabolic pathway gene sets including Prion diseases and Adipocytokine signaling; leptin peptide, an adipocytokine, is known to affect amyloid beta, a major component of amyloid plaques found in the brains of AD patients [23], [24]. The relationship between AD, prion diseases, and adipocytokine signaling pathway reflects the functional dependence at the gene transcriptional level. Except for Citrate cycle (TCA) linked to all three diseases and Neurotrophin signaling pathway connected to Alzheimer's disease, all the other pathway relationships with the three neurodegenerative diseases are unique to the co-DE gene set network. Previous studies [25], [26] have suggested that interactions between pathways are highly context specific. In order to provide further support for the identified co-DE relationships with neurodegenerative diseases and verify that the co-enrichment gene set network construction algorithm could select relevant experiments from a wide variety of experiments, we investigated the PubMed references associated with experiments where the pathways linked to any of the three diseases were co-enriched with the disease (Table S2). Out of a total of 36 PubMed references, 10 were studies directly related to the brain. Another 13 references were meta-studies incorporating brain samples or involved related topics such as aging, mitochondrial diseases and pathways, protein misfolding and aggregation such as amyloidosis, and adipocytokines. While the remaining 13 references were seemingly unrelated, it does not mean that the co-enrichment of the aforementioned pathways in gene lists from these studies were artifacts. For example, one reference involved expression profiling in cutaneous squamous cell carcinoma [27]; the authors of this study identify and discuss Kalirin (also known as huntingtin-associated protein interacting protein), which has been linked to Alzheimer's disease and schizophrenia [28], along with several mitochondrial chain enzymes whose genes are differentially expressed attributing the link with cutaneous squamous cell carcinoma to increased oxidative stress. Additionally, three references were studies in the liver, which has been suggested to be the origin of Alzheimer's disease plaques [29]. Therefore, the literature evidence underlying the experimentally derived gene lists selected by the co-enrichment algorithm supports the potential functional relationships identified between Alzheimer's, Huntington's, and Parkinson's disease and co-differentially expressed pathways.

In addition to the novel pairwise relationships between pathways, the KEGG Pathway co-DE gene set network has differences in network topology as compared to the co-membership gene set network. While the overall degree centrality is correlated (Pearson's correlation coefficient  = 0.8; Figure S2), the outliers highlight key differences. Specifically, Metabolic pathways, Pathways in cancer, and Focal adhesion are the top three outliers with a greater degree centrality in the co-membership gene set network than the co-DE gene set network. The two aggregate pathways, Metabolic pathways and Pathways in cancer, have an expectedly low degree in the co-DE network considering that common genes between the gene sets and each other pathway gene set are removed prior to assessing co-differential expression. Focal adhesion, a cell communication pathway implicated in several cancers [30], has significant crosstalk with 57 other pathways. On the other hand, there are only 18 pathways co-differentially expressed with focal adhesion, suggesting that focal adhesion involves proteins that participate in several pathways but the transcriptional relationship with entire pathways is more specific. The TGF-beta signaling pathway acts as a super-hub in the co-DE gene set network having the largest increase in degree centrality from co-membership to co-DE gene set networks and is co-differentially expressed with 44 pathways while having significant co-membership with only 16 pathways, suggesting that TGF-beta may be a transcriptional master-regulator. For example, the co-DE relationship between TGF-beta signaling and the Circadian rhythm pathway is not found in the co-membership gene set network; a functional dependence between the two pathways has previously been identified [31].

Another topological difference between the co-membership and co-DE gene set networks is that the co-DE gene set network has greater modularity with 12 distinct modules as opposed to only 5 in the co-membership gene set network. Modules are detected by finding clusters within the gene set networks using a graph cut algorithm (see Methods). If there are a significant number (Benjamini-Hochberg corrected p-value ≤0.05 using Fisher's exact test) of pathways belonging to a KEGG Pathway group as defined on their website, we label the module with the group name (Figure 2). For example, a new eight-pathway “Folding, Sorting, and Degradation” module is unique to the co-DE gene set network and contains i) Protein export, ii) N-Glycan biosynthesis, iii) RNA degradation, iv) Spliceosome, v) RNA polymerase, vi) Proteasome, vii) Aminoacyl t-RNA biosynthesis, and viii) Valine, leucine, and isoleucine biosynthesis pathways (Figure 2C). 10 out of the 14 inter-modular edges from the “Folding, Sorting, and Degradation” module are incident to the “Replication and Repair” module indicating that “Folding, Sorting, and Degradation” pathways are transcriptionally separated from other pathways in the absence of “Replication and Repair” pathways. The “Replication and Repair” module in turn is connected to cell cycle-related pathways (Cell Cycle, Oocyte meiosis, and p53 signaling pathway) in the “Signal transduction and Cancers” module with 18 out of 31 edges between the “Replication and Repair” and “Signal transduction and Cancers” modules incident to one of the three cell cycle-related pathways. While the “Replication and Repair” and “Signal transduction and Cancers” modules are also present in the co-membership meta-graph, the focused inter-modular links between the “Folding, Sorting, and Degradation” module and “Replication and Repair” and the hyper-connectedness of cell cycle-related pathways to the “Replication and Repair” module results in a linear organization of pathway modules in the co-DE gene set network in the following order: “Immune System Diseases”, “Signal transduction and Cancers”, “Replication and Repair”, and “Folding, Sorting, and Degradation” (Figure 2B).

### The Chromosome Map co-differential expression gene set network

The human chromosome map is a collection of gene sets where each gene set corresponds to a single chromosome locus as defined by cytogenetic bands. By definition, there cannot be co-membership between any pair of gene sets in the Chromosome Map since the same gene cannot be placed in two loci. The PPI gene set network reveals that there are no physical interaction relationships between chromosome loci. Therefore, the chromosome map co-DE gene set network is an ideal example to demonstrate the utility of the co-enrichment approach.

**Figure 3:**
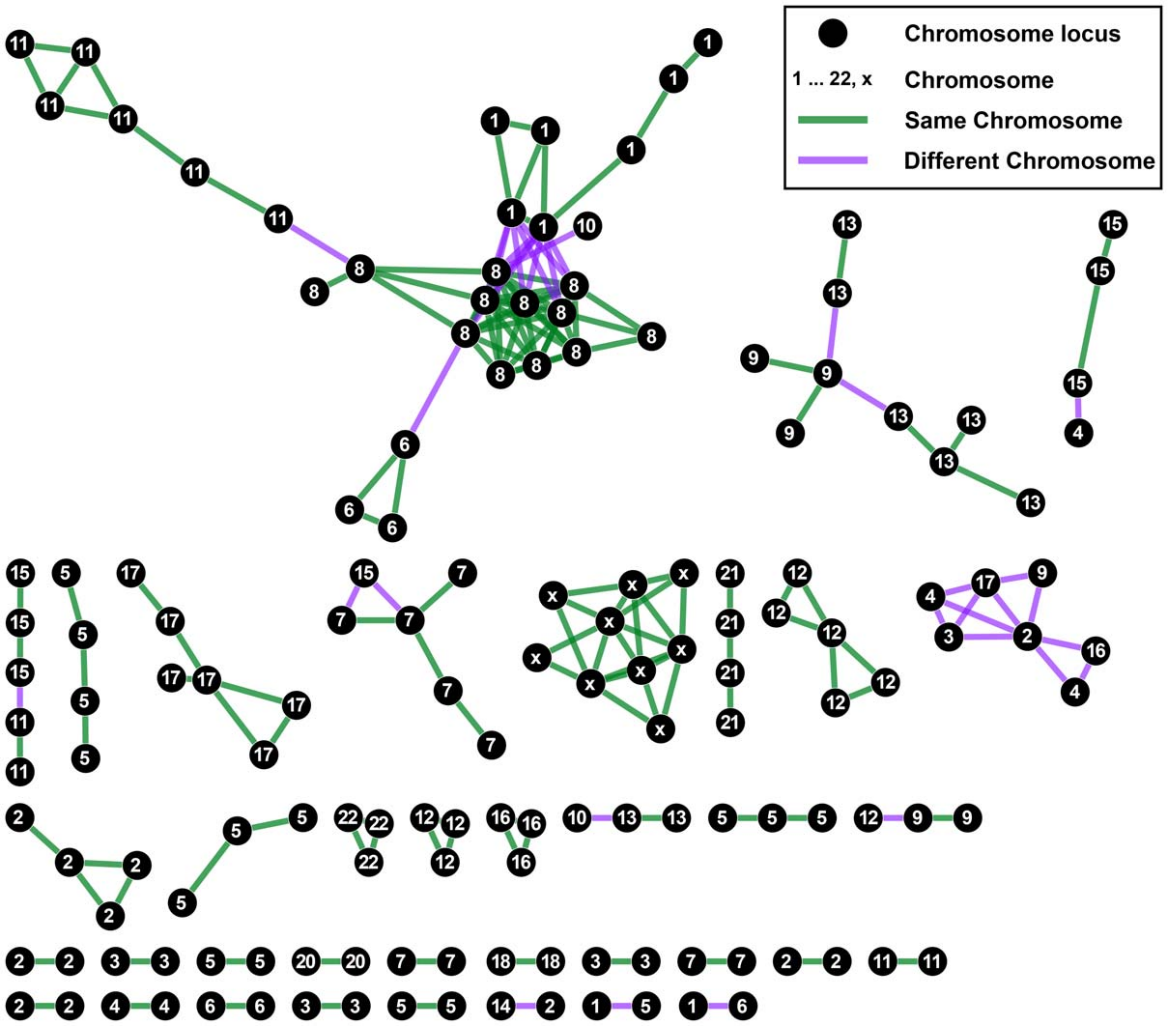
Chromosome Map co-differential expression gene set network. Nodes representing chromosome loci are connected if they are significantly co-differentially expressed. Green edges connect loci on the same chromosome while purple edges connect loci on different chromosomes. The autosomes and x chromosome are indicated within each node.

We find 182 pairs of chromosome loci that are co-differentially expressed across many gene expression experiments (Figure 3). Most of the edges (146 out of 182) connect pairs of loci that are on the same chromosome; the network is enriched for same-chromosome edges (p-value  = 6.57E-159 using Fisher's exact test). Many of the loci pairs are adjacent, such as the most significant edge connecting chr8p11 with chr8p12 (Table 1). While that may lend the hypothesis of large expression domains, it is important to note that these two loci span approximately 16 mega bases (MB). Furthermore there are several edges connecting loci that are non adjacent and are often on different arms or different chromosomes suggesting that linear proximity cannot be the sole explanation for co-differential expression of entire chromosome loci.

**Figure 4:**
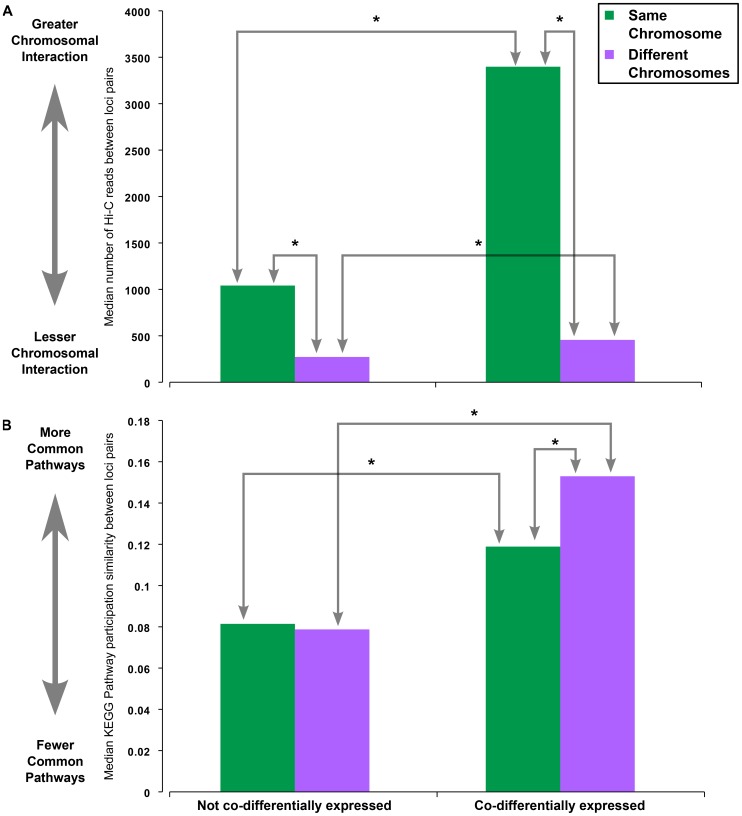
Differences in co-differentially expressed chromosome loci with respect to 3D proximity and pathway participation. Data averages for same chromosome loci pairs are shown as green columns while data averages for different chromosome loci pairs are shown as purple columns. The columns are separated by whether the loci pairs are co-differentially expressed or not. A) The median number of Hi-C reads indicating contacts between pairs of chromosome loci in 3D space. B) The median pathway participation profile similarity between loci pairs computed based on the KEGG Pathway annotations of the corresponding genes. Asterisks (*) indicate a significant difference in values (Benjamini-corrected Wilcoxon-Mann-Whitney test p-value <0.05).

We hypothesized that the co-differential expression of these large sections of the genome might be due to proximity in three-dimensional (3D) space. We collected Hi-C data from Lieberman-Aiden *et al*. [32] that combines chromosome conformation capture (3C) with next-generation sequencing to determine chromosomal contacts in 3D space between pairs of 1 MB regions of the entire human genome (except chromosome Y) in karyotypically normal cells where regions with greater interactions have a greater number of sequenced reads spanning the two 1 MB genomic regions. We summed the reads per chromosome loci pair to include all 1 MB regions within the defined genomic coordinates for the respective loci from UCSC human genome build 18 (same build as the Hi-C study). We observe that pairs of loci on the same chromosome have expectedly and significantly more interactions in 3D space than loci pairs on different chromosomes and that the relative increase is independent of whether the loci pair are in the co-DE gene set network or not (Figure 4A). However, loci pairs that are co-differentially expressed have significantly greater number of contacts than those that are not co-differentially expressed for same chromosome loci pairs (Benjamini-corrected Wilcoxon-Mann-Whitney test p-value  = 1.8E-30) as well as different chromosome loci pairs (Benjamini-corrected Wilcoxon-Mann-Whitney test p-value = 0.003) (Figure 4A).

Three-dimensional proximity only partially explains the co-differential expression of chromosome loci pairs since the number of Hi-C reads for co-differentially expressed pairs on different chromosomes is still significantly less than the number of Hi-C reads for same chromosome loci pairs that are not co-differentially expressed (Benjamini-corrected Wilcoxon-Mann-Whitney test p-value = 0.001. Therefore, we hypothesized that there may be a functional dependence that is not captured solely by genomic organization between loci pairs on different chromosomes that are co-differentially expressed. In order to test whether there may be a functional dependence between pairs of entire chromosome loci, we calculated KEGG Pathway participation similarity between all pairs of loci (see Methods). We find that there is no difference in pathway similarity between pairs of loci on the same chromosome versus different chromosomes for pairs that are not co-differentially expressed (Figure 4B). However, loci pairs in the co-DE gene set network are more similar in terms of KEGG pathway participation than those that are not co-differentially expressed irrespective of whether they are on the same chromosome or not. Notably, the co-differentially expressed loci pairs on different chromosomes have a significantly greater pathway participation profile similarity (Benjamini-corrected Wilcoxon-Mann-Whitney test p-value = 0.045) than co-differentially expressed loci pairs on the same chromosome, a pattern that was not observed in loci pairs that are not co-differentially expressed (Figure 4B).

The relationship between gene co-expression, as determined from concordant gene expression, and 3D proximity has previously been reported [33]. Likewise, the relationship between gene co-expression and pathway participation [34], linear proximity and gene co-expression [35], and linear proximity and pathway participation in eukaryotes [36] has also been previously reported. However, this study, to our knowledge, is the first to report a relationship between inter-chromosomal co-differential expression, 3D proximity, and pathway participation.

### Comparing the Phosphorylation Substrates co-membership and PPI gene set networks

We construct a series of gene sets containing substrates of each of 330 kinases and 59 phosphatases. The co-membership gene set network therefore connects kinases or phosphatases with each other if there is a significant overlap between their substrates (Figure S3). The PPI gene set network connects pairs of phosphorylation enzymes with each other if their unique substrates have a significant number of physical interactions between them (Figure S4). The gene set networks can be used to infer relationships between kinases or phosphatases based on evidence presented by their substrates.

There are a total of 995 edges in the co-membership gene set network with 71.1% of the edges between two kinases, 7.6% of the edges between two phosphatases, and 21.3% of the edges between a kinase and a phosphatase. There is minimal overlap (18 edges; Figure 1D) between the co-membership and PPI gene set network, which consists of 337 edges with 70.6% of the edges between two kinases, 1.8% of the edges between two phosphatases, and 27.6% of the edges between a kinase and a phosphatase. The small overlap and differing distributions for edges incident to a phosphatase suggests that the two gene set networks provide complementary information.

**Figure 5:**
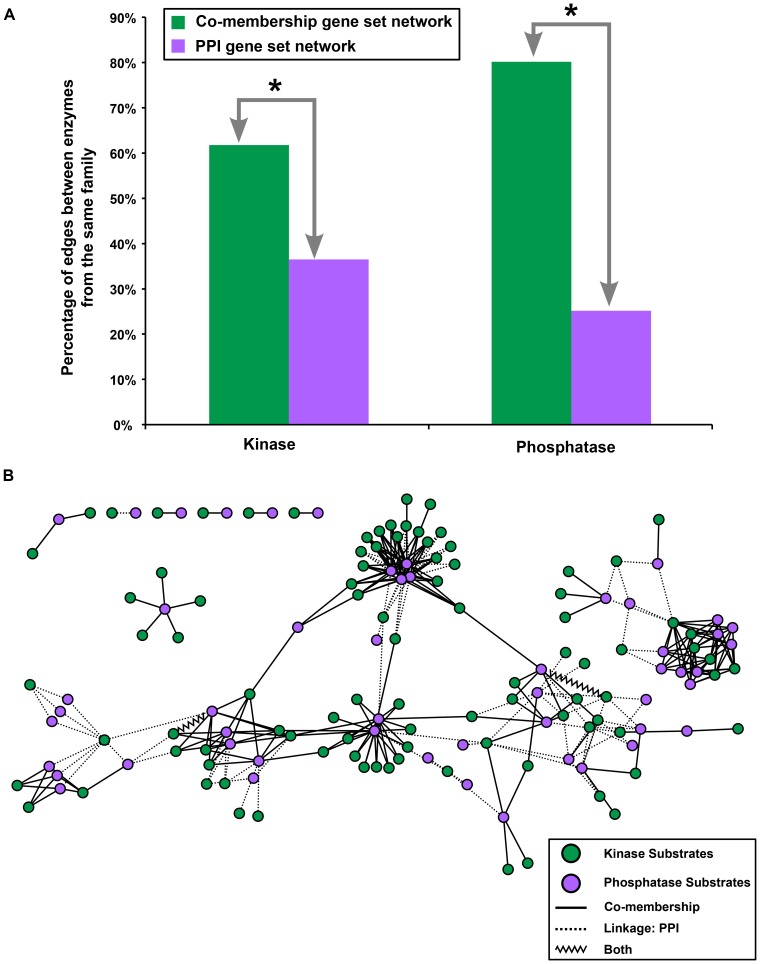
Phosphorylation Substrates gene set networks. A) The percentage of connected kinase or phosphatase pairs in the Phosphorylation Substrates co-membership and PPI gene set networks that belong to the same kinase or phosphatase family respectively are determined based on enzyme-family assignment from the KEGG BRITE and GO databases. The co-membership gene set network has a greater percentage of enzyme pairs from the same family than the PPI gene set network. Asterisks (*) indicate a significant difference in the percentage of same-family pairs (p-value <0.0001, see Methods for calculation details). B) Phosphorylation Substrates co-membership (solid lines) and PPI gene set network edges (dotted lines) between a kinase (green nodes) and a phosphatase (purple nodes). The two kinase-phosphatase edges found in both networks are shown as zigzags.

The co-membership gene set network connects kinases or phosphatases from the respective same family to a significantly greater extent than the PPI gene set network (p-value <0.0001 See Methods, Figure 5A) supporting the notion that related enzymes phosphorylate or dephosphorylate the same substrates. Kinase prediction algorithms based on sequence motif searches alone have been limited to family level resolution [37]. The co-membership gene set network provides evidence that substrate specificity may resolve at the family level and that further specificity may arise from other biological context such as cellular localization [38]. It is important to note that the kinase family assignments are based on sequence similarity of the kinase domains [39] indicating that the kinase domain is responsible for substrate specificity at family level resolution. The phosphatases are separated based on 6 GO molecular function groupings separated by the residue they dephosphorylate under the “phosphoprotein phosphatase activity (GO:0004721)” parent term.

The PPI gene set network is constructed based on physical interactions between substrates. Remarkably, the connected enzymes in the PPI gene set network themselves physically interact to a significantly greater extent (p-value <0.0001, See Methods) than those connected in the co-membership gene set network. 26% of connected enzyme pairs in the PPI gene set network physically interact with each other as opposed to 19% of pairs in the co-membership gene set network; only pairs where both enzymes have at least one reported protein-protein interaction were considered. This result suggests that if the substrates of two kinases or phosphatases physically interact, then the enzymes themselves physically interact.

Figure 5B shows all co-membership and PPI gene set network edges between a kinase and a phosphatase. The gene set network approach thus integrates substrate information to identify cooperative kinase and phosphatase pairs and modules. Upstream kinase prediction algorithms can potentially benefit from the added information about phosphatases that have the same substrate specificity.

### MetaNet web tool

The aforementioned examples demonstrate that gene sets are not isolated biological themes but form vast interconnected networks. The pre-computed gene set networks can facilitate organization of high-throughput data into context-dependent gene set networks. We therefore constructed MetaNet, a tool for connecting over-represented biological themes in user-defined gene lists based on pre-computed gene set networks. The constructed context-specific gene set networks can then be used to generate systems level hypotheses. MetaNet is freely available at http://blaispathways.dfci.harvard.edu/metanet/.

**Figure 6:**
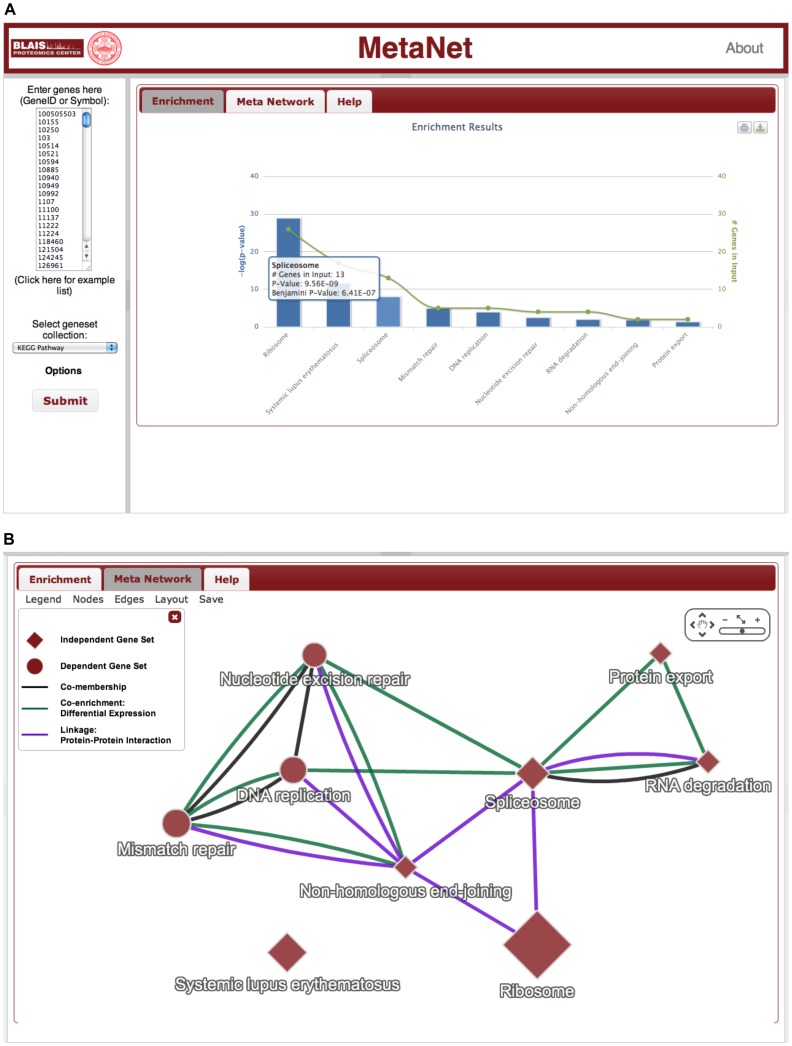
MetaNet web tool. Screenshots of the MetaNet web tool for mapping enriched gene sets in a user-defined gene list onto pre-computed gene set networks. A) Enrichment results reported as an interactive column graph with further details reported upon click or hover on the columns. B) Enriched gene sets connected in a gene set network based on pre-computed co-membership (black), linkage:ppi (blue), and co-enrichment: differential expression (green) edges. Gene sets that have a greater number of unique members than number of members shared with any other gene set are shaped as diamonds. The network is interactive with controls for node/edge format, graph layout, and graph export (PNG, PDF, SIF, and graphML) options. Additional information about the nodes and edges is displayed on click.

As a use case, we analyzed a list of proteins identified as components of the Ku complex from a nuclear fraction of HeLa S3 cells using tandem affinity purification (TAP) coupled with mass spectrometry [40]. The proteins were converted to Entrez Gene IDs using the UniProt ID Mapping service [41] and submitted to MetaNet to be analyzed for enrichment of KEGG Pathways. The MetaNet tool identifies DNA damage, transcription, and ribosome assembly pathways (Figure 6A), a finding that is consistent with that reported by the authors of the study. However, a list of overrepresented pathways does not provide insight into the relationship between the pathways.

The MetaNet tool constructs a gene set network of the enriched gene sets with edges from all pre-computed co-membership, linkage, and co-enrichment gene set networks. The current version of MetaNet contains co-membership, PPI and co-differential expression gene set networks. The KEGG Pathways that are enriched amongst the Ku complex genes are connected in a gene set network with multiple edges from the aforementioned pre-computed gene set networks (Figure 6B). The constructed context-specific gene set network shows that only Systemic lupus erythematosus, which is enriched due to a high number of histones annotated as belonging to the pathway, is functionally disjoint; i.e. every other enriched pathway is connected to at least one other enriched pathway. There are a total of 20 edges between 9 enriched pathways with 9 co-differential expression edges, 7 PPI edges, and 4 co-membership edges. The 7 PPI edges confirm a scaffold of interacting complexes and pathway components that comprise the entire Ku complex. The Ribosome gene set is linked to the Spliceosome and Non-homologous end-joining pathways via physical interaction alone; indeed the authors suggest that ribosomal proteins may be recruited by the Ku complex at the site mRNA synthesis for regulation of translation. A feature of the MetaNet context-specific gene set network is that gene sets are highlighted if the number of unique genes also in the user-input is greater than the number of shared genes with any other gene set (diamond nodes in Figure 6B); we chose to highlight context-specific nodes instead of adding context-specific edges to the pre-computed ones in order to provide a positive user experience by avoiding lengthy on-the-fly computations and cluttered graphs. In this case, all KEGG Pathway gene sets except DNA replication, Mismatch repair, and Nucleotide excision repair have a greater number of unique representative genes in the input list than shared genes. DNA replication, Mismatch repair, and Nucleotide excision repair gene sets form a 3-node co-membership clique, which explains why the pathways are co-dependent in this example.

All MetaNet results can be downloaded as graphics or text files; the gene set network graph visualization can be downloaded in PDF and PNG and the graph contents and structure can be download in SIF or GraphML format that can be subsequently imported in visualization tools. While MetaNet is not designed to visualize global gene set networks, all pre-computed gene set networks in their entirety and all gene set annotations are available for download in tab-delimited text format, which can be imported into network visualization tools such as Cytoscape [12]. Gene set databases are continually updated with new or modified annotations of genes as well as new gene sets. As a balance between incorporating gene set updates and reproducible data analysis, we will update the MetaNet database with recomputed gene set networks from updated gene set annotations on an annual basis. The source code is stored in a Git repository and is available through the MetaNet Help page.

## Discussion

Gene set networks map functional relationships between biological themes and can be used to identify novel insights into their organization. While co-membership gene set networks intuitively link similar gene sets, based on shared entities, they cannot be used where the expected overlap between any pair is negligible as in the case of chromosome loci. Co-enrichment and co-linkage gene set networks integrate additional evidence with curated gene sets to identify hidden relationships. To our knowledge, this is the first reported study describing the co-enrichment method for gene set network construction. The advantage of this approach is that the experimental data are included as gene lists, allowing independent instrument and experiment specific processing of the data. Thus, differentially expressed genes lists from next-generation sequencing data can be readily integrated for the construction of co-differential expression gene set networks using the co-enrichment method. Given the flexibility in processing, the approach can be extended to other types of data. For example, co-differential expression is not an appropriate measure of functional relationship between the Phosphorylation Substrates collection of gene sets. In this case, phosphoprotein lists derived from phosphoproteomics experiments can replace differentially expressed gene lists. We tested the approach on protein lists derived from proteomics studies from the PLIPS database [42] to construct a KEGG Pathway proteomic study co-occurrence gene set network (Figure S5). However, unlike lists generated from gene expression microarray studies that present differentially expressed genes, lists generated from proteomics measurements may not always indicate a differential measurement. Consistently interpretable protein lists would be needed to be able to use proteomics or phosphoproteomics data to generate meaningful gene set networks.

A feature of the co-enrichment and linkage gene set network construction algorithms is that the removal of shared genes penalizes highly overlapping pairs of gene sets and thusly facilitates identification of novel relationships between gene sets. However, in the case of GO gene sets there are trivial and uninformative subset relationships, resulting from the hierarchical GO DAG. Methods such as GO slim [3] select a handful of broad terms from distinct branches of the GO DAG in order to avoid redundant terms. Here we use a filtering approach (see Methods) to select non-redundant and informative GO terms from distinct lineages of the GO DAG with a parameter dictating the expected cardinality of each GO gene set. Unlike GO slim, which is widely adopted, the GO terms selected by our approach may not overlap with terms identified using other tools, making it difficult to integrate the GO gene set networks described here with GO terms identified using tools other than MetaNet. The GO terms selected and consequently the gene set networks constructed using them will differ based on the cardinality parameters of our filtering approach. However, we find that the filtering is relatively robust for moderate changes to the parameters. For example, changing the expected cardinality from 50 to 100 retains 76%, 87%, and 74% of the same gene sets and 40%, 62%, and 35% of the same co-membership edges for biological process, molecular function, and cellular component namespaces respectively (File S2). Our filtering approach does not explicitly account for imbalances in the GO DAG such as the relative breadth and depth of lineages; while our method reduces redundancies by removing parents and children in the GO DAG, further filtering of terms from especially broad lineages would reduce the number of redundant sibling terms.

All three gene set network construction methods require N*(N-1)/2 computations corresponding to all pairs of N gene sets in a collection. The co-enrichment method requires additional M computations at each of the N*(N-1)/2 steps where M is the number of experimentally derived gene lists to be tested for enrichment of gene sets. While there may be ways to reduce the number of computations, current computing power along with parallel processing easily allow all pairwise comparisons of gene sets from popular databases.

## Methods

### Data sources

KEGG Pathway annotations (release: 55.1; download/last modified date: 09-13-2010) [4] were downloaded from the KEGG FTP server. The chromosome map was downloaded from MSigDB (version 2.5; download/last modified date: 09-14-2010) [43]. The GO annotations (GOA CVS version 1.189; download/last modified date: 04-18-2011) and tree were downloaded from the Gene Ontology website. VirusMINT annotations (download/last modified date: 09-22-2010) were made available at the VirusMINT website. The kinase-substrate and phosphatase-substrate relationships that compose the Phosphorylation Substrates gene sets were compiled from Human Protein Reference Database (HPRD) (version 8; download/last modified date: 07-06-2009) [44], KEGG Pathway, and PhosphoSitePlus (download/last modified date: 03-11-2010) [45]. In all cases, gene sets within a collection that were exactly identical in their membership were combined. Protein-protein interaction (PPI) data for the construction of the PPI gene set networks were collected from HPRD, KEGG Pathway, and BioGRID (version 3.1.70; download/last modified date: 10-26-2010) [46] databases. A total of 5177 lists of differentially expressed (DE) genes were collected from L2L (953 lists) (version 2007.1; download/last modified date: 06-25-2007) [47], CCancer (3266 lists) (download/last modified date: 06-10-2010) [48], and GeneSigDB (958 lists) (release 2; download/last modified date: 03-05-2010) [49] for the construction of co-differential expression gene set networks. It is important to note that there is a bias towards cancer related experiments in each of the three aforementioned DE gene list databases.

### Gene Ontology filtering

GO gene sets per namespace (biological process, molecular function, and cellular component) were selected such that each term had a cardinality that was closest to 50 within its lineage; i.e. for each term none of its predecessor or successor terms in the GO DAG had a cardinality closer to 50 than its own cardinality and were thus removed. A similar method has been previously used by Linghu *et* *al.* to select a reduced set of informative GO terms [1]. The choice of 50 as cardinality parameter was chosen since the average cardinality for all the non-GO gene sets in this study was 45.5. Additionally, GO gene sets with cardinality less than 10 or greater than 200 were removed. Setting an upper and lower bound on GO gene set cardinality is a common method [5], [8], [50] for selecting terms that are neither too specific nor too broad.

### Constructing a co-membership gene set network

A co-membership gene set network for a specified collection of gene sets such as KEGG Pathways connects a pair of gene sets if there are a significant number of genes in common. The procedure is as follows:

1. For a given pair of gene sets in a gene set collection count the number of genes in one gene set, the number of genes in the other gene set, and the number of overlapping genes.
2. Calculate a p-value for significantly high number of overlapping genes using Fisher's exact test. The background for the test is the set of all genes present in any gene set in the collection.
3. Repeat for every unique pair of gene sets and adjust calculated p-values for multiple hypotheses using the Benjamini-Hochberg method.
4. Connect a pair of gene sets if the Benjamini-Hochberg corrected p-value ≤0.05.

### Constructing a linkage gene set network

A linkage gene set network for a specified collection of gene sets such as KEGG Pathways connects a pair of gene sets if there are a significant number of edges in a reference single-biomolecule network, such as a protein-protein interaction network, that link the unique genes of one pathway with the unique genes of the other pathway. The procedure is as follows:

1. Remove all nodes from the reference single-biomolecule network corresponding to genes that are not present in any gene set in the gene set collection.
2. For a given pair of gene sets in a gene set collection remove all shared genes from both gene sets.
3. Count the number of edges, in the reference single-biomolecule network, that link genes from one gene set to any gene, the number of edges that link genes from the other gene set to any gene, and the number of edges that link genes from one gene set to those in the other gene set.
4. Calculate a p-value for significantly high number of edges linking genes from one gene set to those in the other gene set using Fisher's exact test. The background for the test is the set of all edges in the reference single-biomolecule network.
5. Repeat for every unique pair of gene sets and adjust calculated p-values for multiple hypotheses using the Benjamini-Hochberg method.
6. Connect a pair of gene sets if the Benjamini-Hochberg corrected p-value ≤0.05.

### Constructing a co-enrichment gene set network

A co-enrichment gene set network for a specified collection of gene sets such as KEGG Pathways connects a pair of gene sets if there are a significant number of experimentally derived gene lists from a reference set of experiments, such as gene expression microarray experiments, where both gene sets are significantly overrepresented. The procedure is as follows:

1. For a given pair of gene sets in a gene set collection remove all shared genes from both gene sets.
2. Calculate a p-value for significant enrichment of all gene sets, including the pair of gene sets whose shared genes have been removed, in every experimentally derived gene list using Fisher's exact test.
3. Count the number of experimentally derived gene lists where either of the two gene sets from step 1 are significantly enriched, having a Benjamini-Hochberg corrected p-value ≤0.05.
4. Count the number of experimentally derived gene lists where both gene sets from step 1 are significantly enriched.
5. Calculate a p-value for significantly high number of experimentally derived gene lists where both gene sets from step 1 are enriched using Fisher's exact test. The background for the test is the set of all experimentally derived gene lists.
6. Repeat for every unique pair of gene sets and adjust calculated p-values for multiple hypotheses using the Benjamini-Hochberg method.
7. Connect a pair of gene sets if the Benjamini-Hochberg corrected p-value ≤0.05.

### Finding clusters in gene set networks

GRACLUS [51] was used to identify clusters within the gene set networks. The graph is first bisected resulting in two clusters. Each cluster is then recursively split if the subgraph density, calculated as 2E/[V*(V−1)] where E is the number of edges and V is the number of nodes, for each of the two child cluster is greater than the density of the parent cluster. An additional constraint that a cluster must have at least 5 vertices is also applied.

### KEGG Pathway participation similarity for pairs of chromosome loci

For two loci gene sets gs_i_ and gs_j_, we construct two sets, KP_i_ and KP_j_ containing the KEGG pathways that genes in gs_i_ and gs_j_ participate in respectively. The Jaccard similarity coefficient between KP_i_ and KP_j_ is used as the KEGG Pathway participation similarity metric.

### P-value calculation for comparing Phosphorylation Substrates co-membership and PPI gene set networks

We generated 10,000 pairs of random networks by randomizing both the co-membership and PPI Phosphorylation Substrates gene set networks using the edge-swap method (1000 swaps per network) [52]. The p-values were calculated directly from the null distributions generated from the random networks. For calculating the p-value for increase in percentage of edges belonging to the same family, we generated a null distribution of absolute differences in percentage same family between the co-membership and PPI random gene set networks for kinases and phosphatases separately. Similarly, for calculating the p-value for increase in percentage of enzyme pairs that also physically interact, we generated a null distribution of absolute differences in percentage of enzyme pairs that also interact between the co-membership and PPI random gene set networks.

### MetaNet web tool implementation

MetaNet was developed using Python 2.6, Django 1.3.1, and jQuery 1.8.6. The underlying server software is Apache 2.2. Graphs are visualized using the Cytoscape Web plugin [53]. The web tool is supported on Chrome, Firefox, and Safari browsers.

## Supporting Information

Figure S1 **KEGG Pathway neurodegenerative diseases co-differential expression sub-network.** Metabolic pathways are in green, non-metabolic pathways are in purple, and the three disease pathways are marked as diamonds.
(PDF)

Figure S2 **KEGG Pathway co-membership versus co-differential expression gene set network degree centrality.** Each dot indicates a particular pathway. The two aggregate pathways, Metabolic Pathways and Pathways in cancer, are highlighted as large circles. Pathways that have the highest difference in degree centrality relative to either gene set network are also highlighted as large circles.
(PDF)

Figure S3 **Phosphorylation Substrates co-membership gene set network.** Each node is a kinase (green) or phosphatase (purple) and edges connect enzymes if there is a significant overlap in the substrates they modify.
(PDF)

Figure S4 **Phosphorylation Substrates PPI gene set network.** Each node is a kinase (green) or phosphatase (purple) and edges connect enzymes if there are a significant number of physical interactions between the unique substrates they modify.
(PDF)

Figure S5 **KEGG Pathway proteomics study co-occurrence gene set network.** Nodes represent KEGG pathways; metabolic pathways are in green and non-metabolic pathways are in purple. Edges connect pathways if there are a significant number of protein lists from proteomics experiments where the unique components of both pathways are enriched.
(PDF)

File S1 **Archived gene set networks.** A zip archive of tab-delimited text files containing edges and associate p-values per gene set network.
(ZIP)

File S2 **Archived GO co-membership gene set networks using an expected cardinality threshold of 100.** A zip archive of tab-delimited text files containing co-membership gene set network nodes, edges, and associate p-values per GO namespace.
(ZIP)

Table S1 **Relationship between the number (or percentage) of shared genes and edge presence in a gene set network.** All edges per gene set network are divided into two groups: those with lower or higher number (or percentage) of shared genes than the mean number (or percentage) of shared genes for all possible overlapping gene set pairs.
(PDF)

Table S2 **PubMed evidence for neurodegenerative disease co-differential expression.** PubMed references describing microarray experiments that generated gene lists where KEGG pathways are co-enriched with Alzheimer's, Parkinson's, and Huntington's disease KEGG pathways.
(PDF)
