# Supplementary figures and images for: Multi-Edge Gene Set Networks Reveal Novel Insights into Global Relationships between Biological Themes
Source: PLoS One. 2012 Sep 13;7(9):e45211. doi: 10.1371/journal.pone.0045211 (PMC3441533; doi:10.1371/journal.pone.0045211)

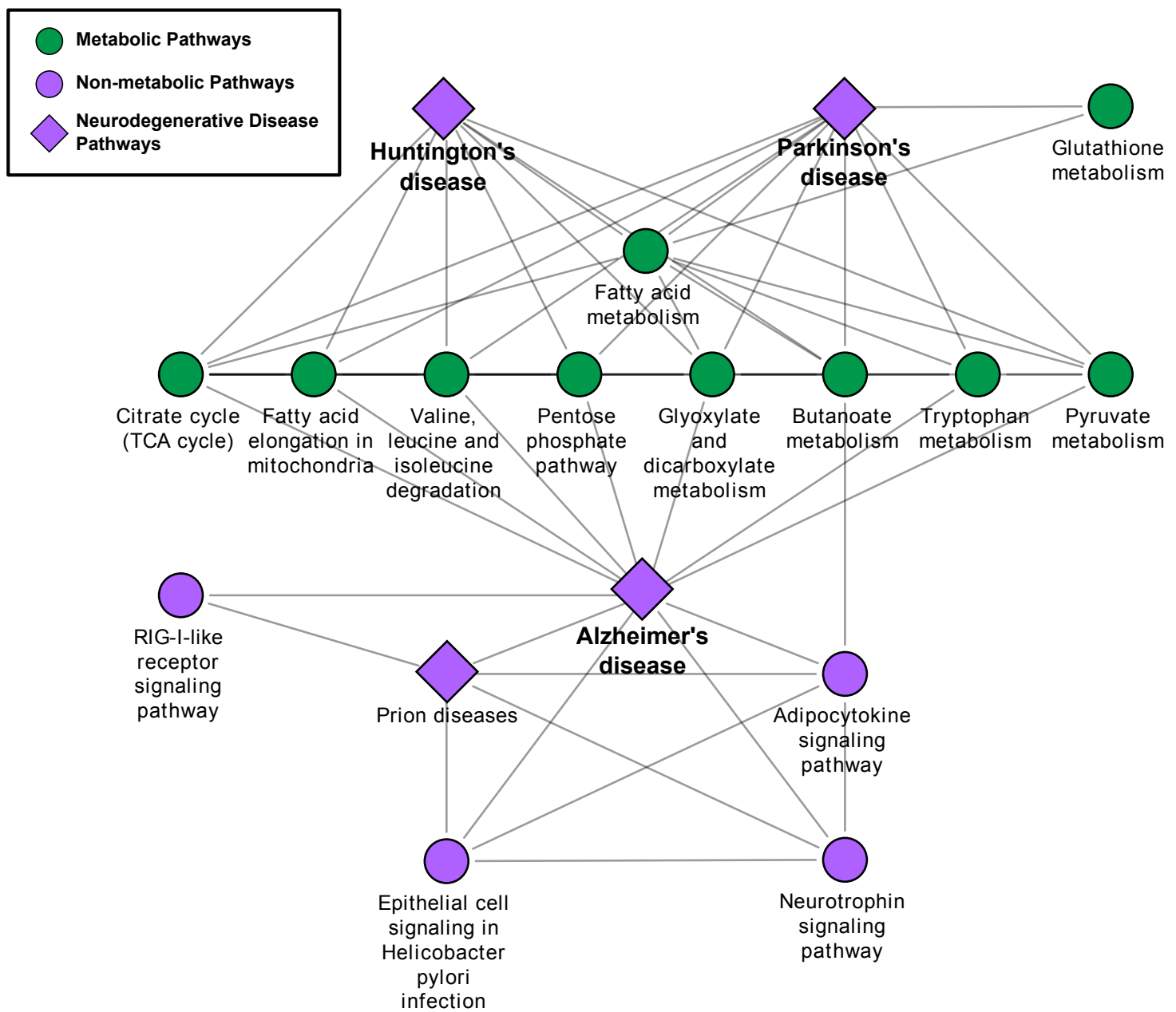

**Supplementary Figure 1**

Supplement: Figure S1 — KEGG Pathway neurodegenerative diseases co-differential expression sub-network. Metabolic pathways are in green, non-metabolic pathways are in purple, and the three disease pathways are marked as diamonds. (PDF) [file pone.0045211.s001.pdf]

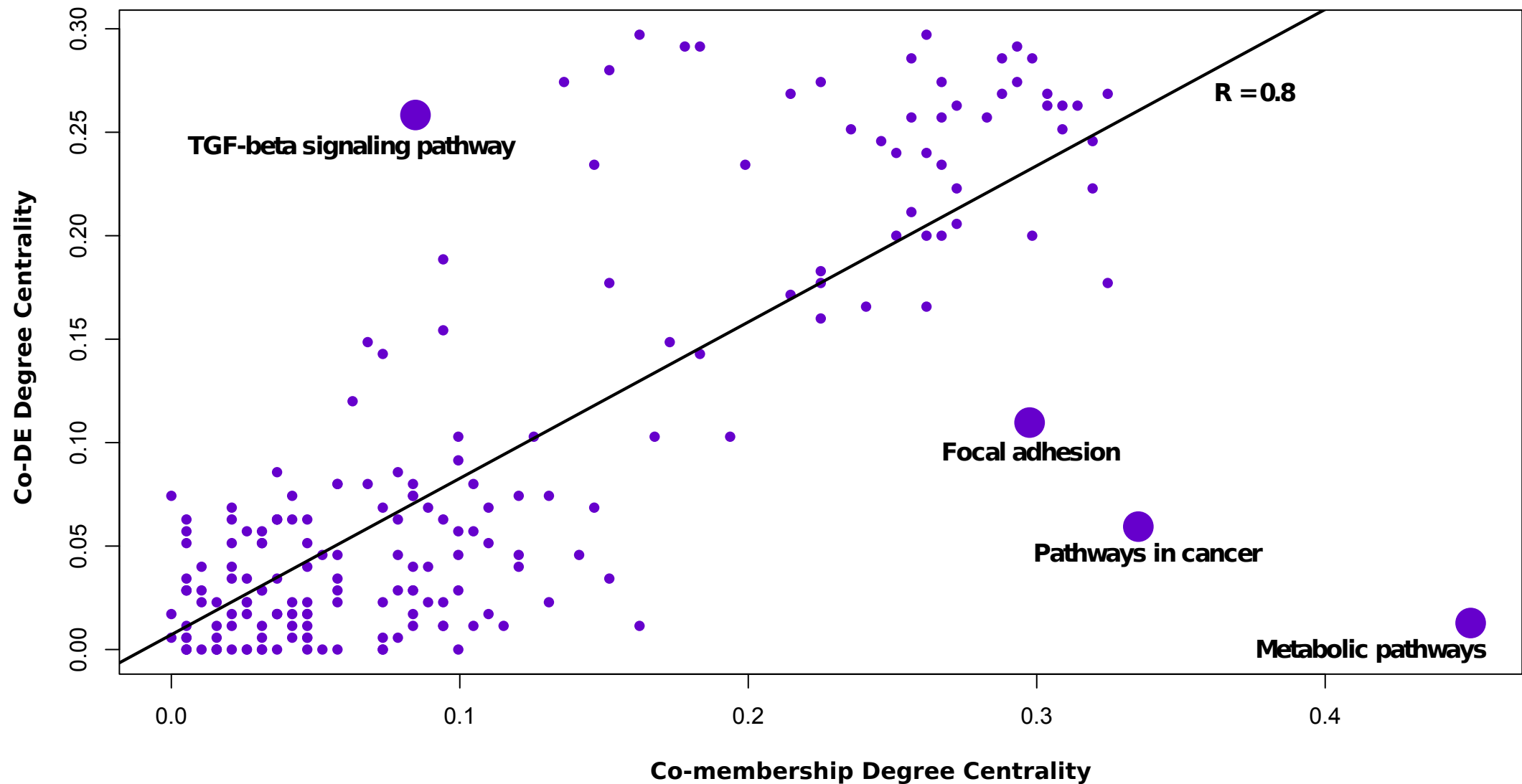

Supplementary Figure 2

Supplement: Figure S2 — KEGG Pathway co-membership versus co-differential expression gene set network degree centrality. Each dot indicates a particular pathway. The two aggregate pathways, Metabolic Pathways and Pathways in cancer, are highlighted as large circles. Pathways that have the highest difference in degree centrality relative to either gene set network are also highlighted as large circles. (PDF) [file pone.0045211.s002.pdf]

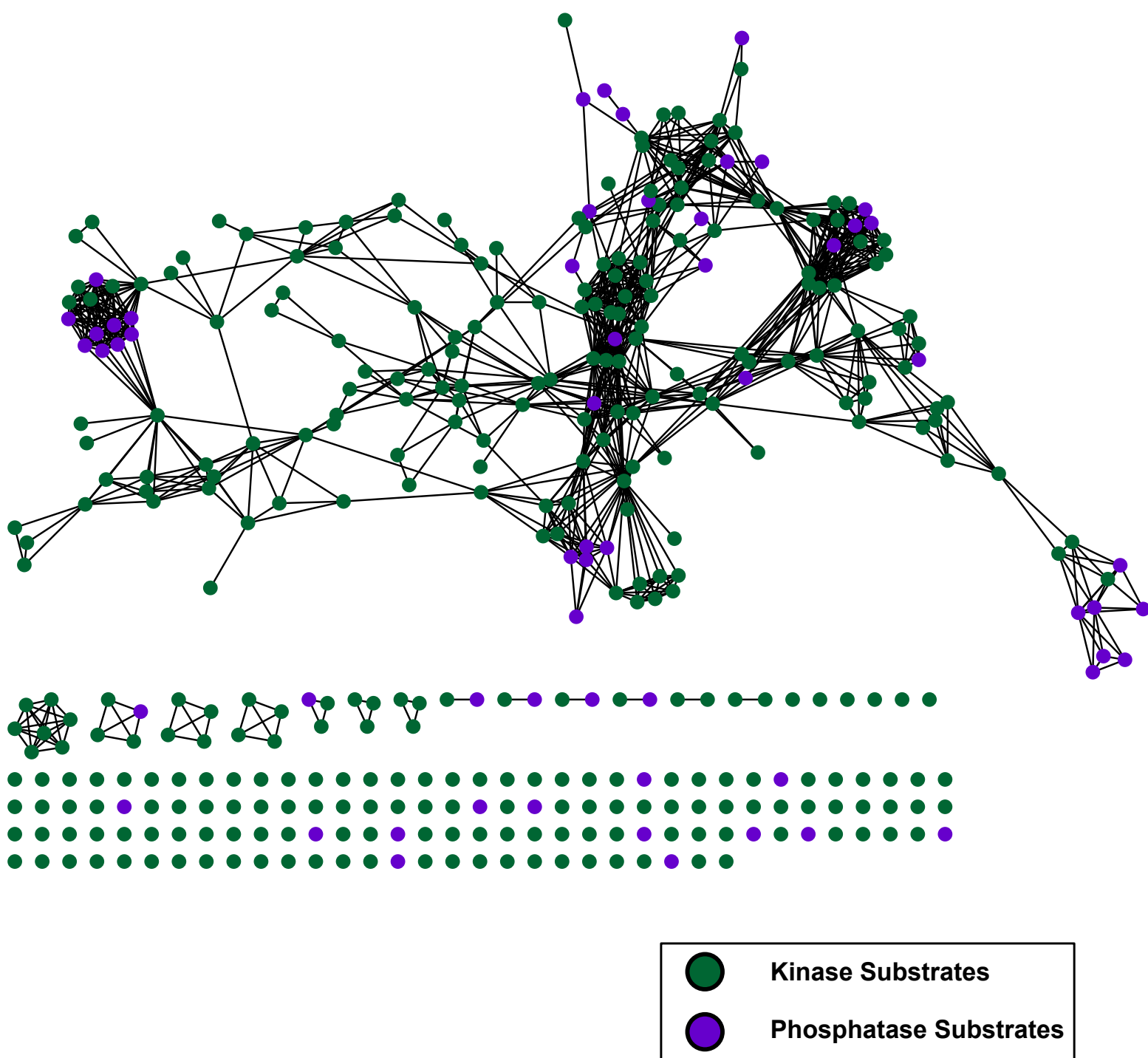

**Supplementary Figure 3**

Supplement: Figure S3 — Phosphorylation Substrates co-membership gene set network. Each node is a kinase (green) or phosphatase (purple) and edges connect enzymes if there is a significant overlap in the substrates they modify. (PDF) [file pone.0045211.s003.pdf]

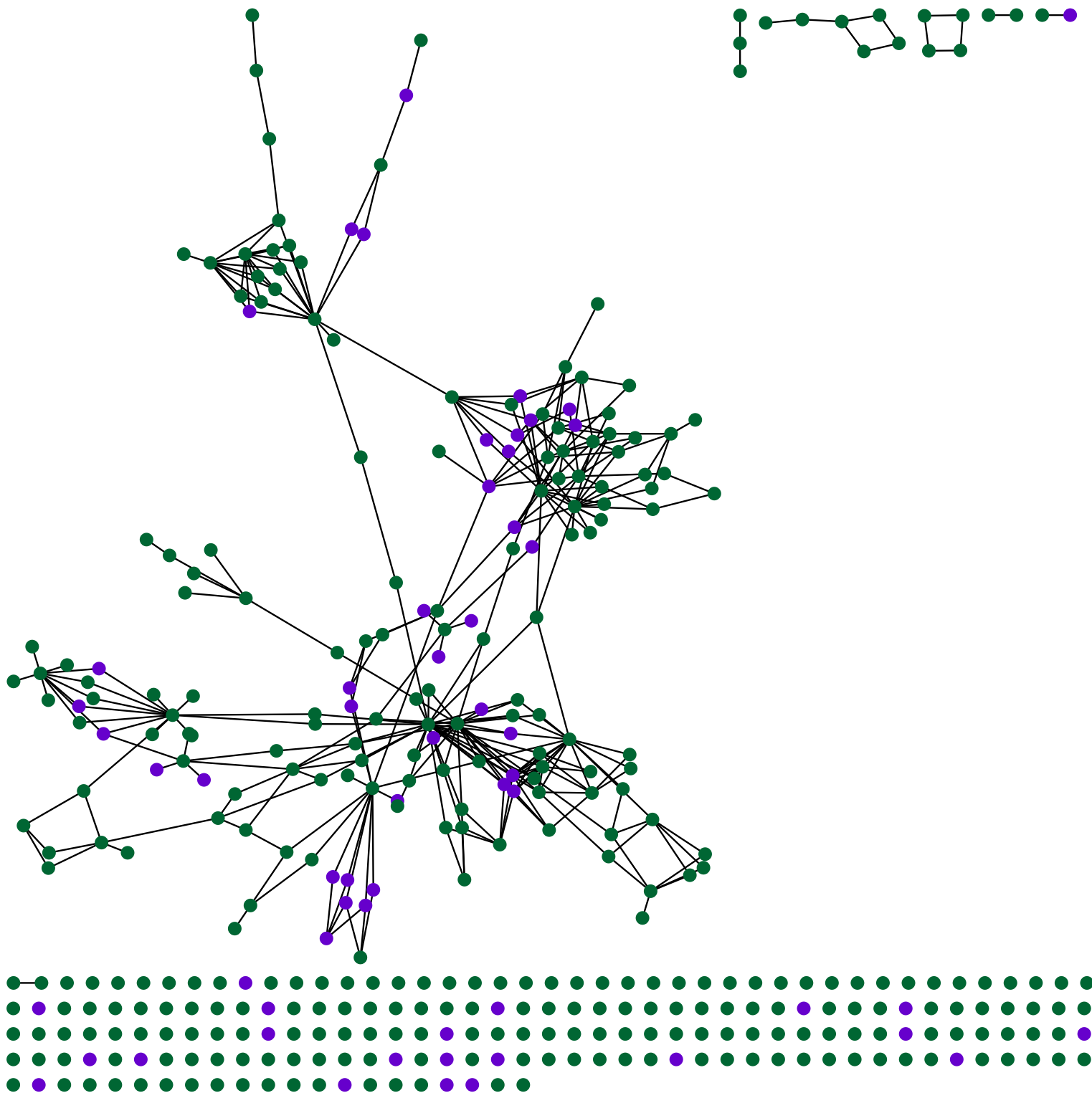

**Supplementary Figure 4**

Supplement: Figure S4 — Phosphorylation Substrates PPI gene set network. Each node is a kinase (green) or phosphatase (purple) and edges connect enzymes if there are a significant number of physical interactions between the unique substrates they modify. (PDF) [file pone.0045211.s004.pdf]

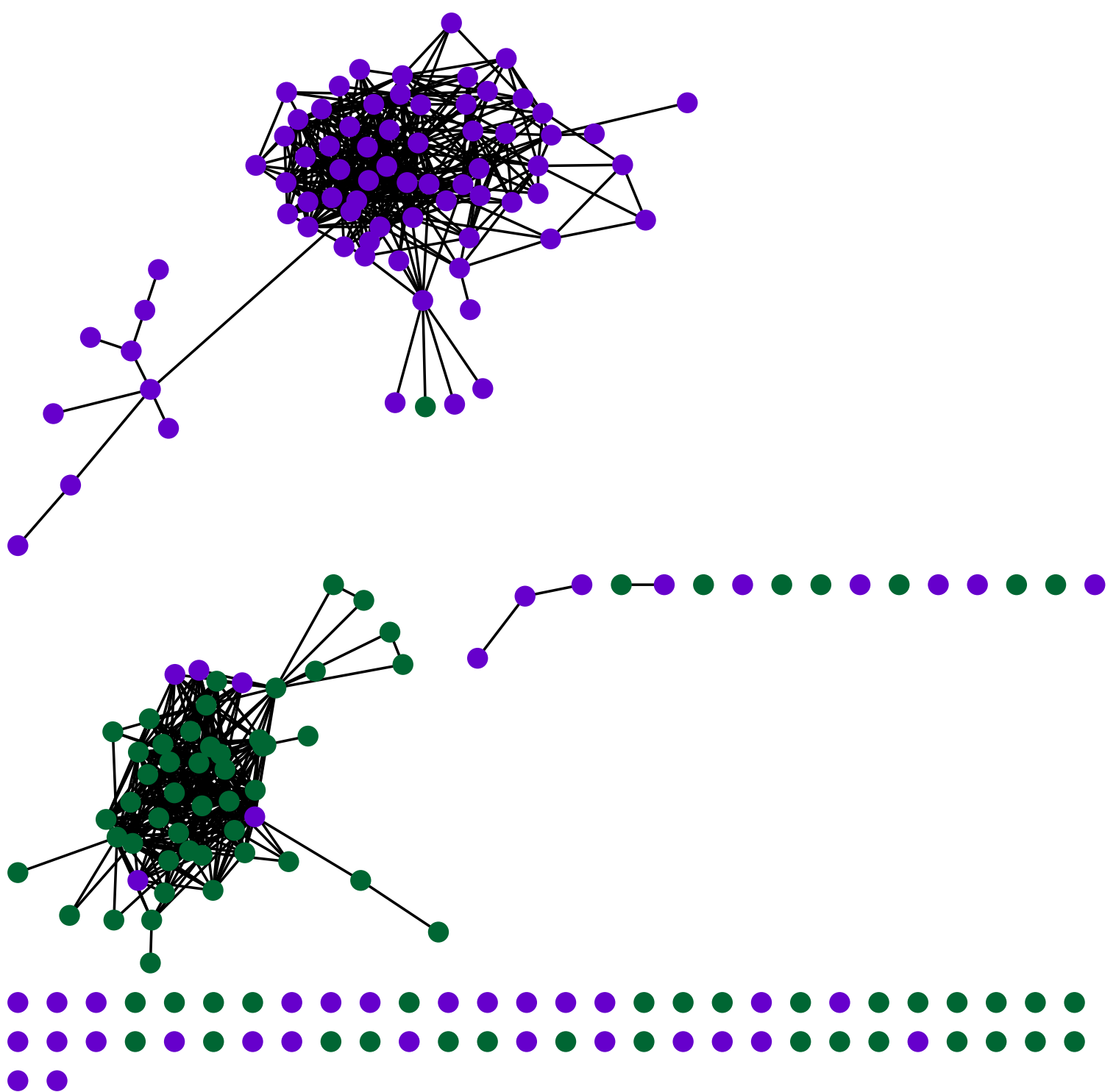

**Supplementary Figure 5**

Supplement: Figure S5 — KEGG Pathway proteomics study co-occurrence gene set network. Nodes represent KEGG pathways; metabolic pathways are in green and non-metabolic pathways are in purple. Edges connect pathways if there are a significant number of protein lists from proteomics experiments where the unique components of both pathways are enriched. (PDF) [file pone.0045211.s005.pdf]
