# Supplementary material for: Multi-Edge Gene Set Networks Reveal Novel Insights into Global Relationships between Biological Themes
Source: PLoS One. 2012 Sep 13;7(9):e45211. doi: 10.1371/journal.pone.0045211 (PMC3441533; doi:10.1371/journal.pone.0045211)
Supplement: Table S1 — Relationship between the number (or percentage) of shared genes and edge presence in a gene set network. All edges per gene set network are divided into two groups: those with lower or higher number (or percentage) of shared genes than the mean number (or percentage) of shared genes for all possible overlapping gene set pairs. (PDF) [file pone.0045211.s008.pdf]

Network	Type	Mean Overlap (Percentage)	Lower than Mean Overlap (Percentage)	Higher than Mean Overlap (Percentage)	Percent Higher Than Mean Overlap (Percentage)	Mean Overlap (Number)	Lower than Mean Overlap (Number)	Higher than Mean Overlap (Number)	Percent Higher Than Mean Overlap (Percentage)
biological_process_50_10_200	coenrichment_differential_expression	7.23%	1395	457	24.68%	1.646016	1324	528	28.51%
cellular_component_50_10_200	coenrichment_differential_expression	9.03%	59	22	27.16%	1.867211	50	31	38.27%
Chromosome_map	coenrichment_differential_expression	0.00%	182	0	0.00%	0	182	0	0.00%
KEGG	coenrichment_differential_expression	19.25%	1033	523	33.61%	10.490748	976	580	37.28%
Kinase_substrates__Phosphatase_substrates	coenrichment_differential_expression	29.97%	50	0	0.00%	2.49127	47	3	6.00%
molecular_function_50_10_200	coenrichment_differential_expression	9.96%	62	19	23.46%	2.374667	60	21	25.93%
WikiPathways	coenrichment_differential_expression	15.34%	585	396	40.37%	5.68078	609	372	37.92%
				**Average**	**21.32%**			**Average**	**24.84%**
biological_process_50_10_200	comembership	7.23%	0	1261	100.00%	1.646016	0	1261	100.00%
cellular_component_50_10_200	comembership	9.03%	0	128	100.00%	1.867211	0	128	100.00%
KEGG	comembership	19.25%	803	1294	61.71%	10.490748	842	1255	59.85%
Kinase_substrates__Phosphatase_substrates	comembership	29.97%	141	854	85.83%	2.49127	111	884	88.84%
molecular_function_50_10_200	comembership	9.96%	0	140	100.00%	2.374667	3	137	97.86%
VirusMINT	comembership	51.21%	19	36	65.45%	2.58589	8	47	85.45%
WikiPathways	comembership	15.34%	155	672	81.26%	5.68078	228	599	72.43%
				**Average**	**84.89%**			**Average**	**86.35%**
biological_process_50_10_200	linkage_ppi	7.23%	179	43	19.37%	1.646016	176	46	20.72%
cellular_component_50_10_200	linkage_ppi	9.03%	92	22	19.30%	1.867211	89	25	21.93%
KEGG	linkage_ppi	19.25%	365	116	24.12%	10.490748	333	148	30.77%
Kinase_substrates__Phosphatase_substrates	linkage_ppi	29.97%	284	53	15.73%	2.49127	281	56	16.62%
molecular_function_50_10_200	linkage_ppi	9.96%	164	34	17.17%	2.374667	167	31	15.66%
VirusMINT	linkage_ppi	51.21%	31	1	3.13%	2.58589	25	7	21.88%
WikiPathways	linkage_ppi	15.34%	311	152	32.83%	5.68078	313	150	32.40%
				**Average**	**18.81%**			**Average**	**22.85%**
