# Supplementary material for: Multi-Edge Gene Set Networks Reveal Novel Insights into Global Relationships between Biological Themes
Source: PLoS One. 2012 Sep 13;7(9):e45211. doi: 10.1371/journal.pone.0045211 (PMC3441533; doi:10.1371/journal.pone.0045211)
Supplement: Table S2 — PubMed evidence for neurodegenerative disease co-differential expression. PubMed references describing microarray experiments that generated gene lists where KEGG pathways are co-enriched with Alzheimer's, Parkinson's, and Huntington's disease KEGG pathways. (PDF) [file pone.0045211.s009.pdf]

Brain Related?	Title	Author	Journal/Year	PubMed ID
Directly related	Soluble epoxide inhibition is protective against cerebral ischemia via vascular and neural protection.	Simpkins AN et al.	Am J Pathol. 2009	PMID:19435785
Directly related	Genomic deletions correlate with underexpression of novel candidate genes at six loci in pediatric pilocytic astrocytoma.	Potter N et al.	Neoplasia. 2008	PMID:18670637
Directly related	A genetic association analysis of cognitive ability and cognitive ageing using 325 markers for 109 genes associated with oxidative stress or cognition.	Harris SE et al.	BMC Genet. 2007	PMID:17601350
Directly related	The commonality of protein interaction networks determined in neurodegenerative disorders (NDDs).	Limviphuvadh V et al.	Bioinformatics. 2007	PMID:17553855
Directly related	Adaptation of energy metabolism in breast cancer brain metastases.	Chen EI et al.	Cancer Res. 2007	PMID:17308085
Directly related	Biomarker discovery: a proteomic approach for brain cancer profiling.	Khalil AA. et al.	Cancer Sci. 2007	PMID:17233837
Directly related	IFN-beta down-regulates the expression of DNA repair gene MGMT and sensitizes resistant glioma cells to temozolomide.	Natsume A et al.	Cancer Res. 2005	PMID:16140920
Directly related	Gene regulation and DNA damage in the ageing human brain.	Lu T et al.	Nature. 2004	PMID:15190254
Directly related	Incipient Alzheimer's disease: microarray correlation analyses reveal major transcriptional and tumor suppressor responses.	Blalock EM et al.	Proc Natl Acad Sci U S A. 2004	PMID:14769913
Directly related	Genome-wide gene expression profiles of the developing mouse hippocampus.	Mody M et al.	Proc Natl Acad Sci U S A. 2001	PMID:11438693
Indirectly related	Integrative analysis reveals the direct and indirect interactions between DNA copy number aberrations and gene expression changes.	Lee H et al.	Bioinformatics. 2008	PMID:18263644
Indirectly related	Transcriptional and phenotypic comparisons of Ppara knockout and siRNA knockdown mice.	De Souza AT et al.	Nucleic Acids Res. 2006	PMID:16945951
Indirectly related	Systematic identification of human mitochondrial disease genes through integrative genomics.	Calvo S et al.	Nat Genet. 2006	PMID:16582907
Indirectly related	Adaptation and increased susceptibility to infection associated with constitutive expression of misfolded SP-C.	Bridges JP et al.	J Cell Biol. 2006	PMID:16449190
Indirectly related	Differentiation stage-dependent preferred uptake of basolateral (systemic) glutamine into Caco-2 cells results in its accumulation in proteins with a role in cell-cell interaction.	Lenaerts K et al.	FEBS J. 2005	PMID:15978041
Indirectly related	Analysis of double-stranded RNA-induced apoptosis pathways using interferon-response noninducible small interfering RNA expression vector library.	Matsumoto S et al.	J Biol Chem. 2005	PMID:15845550
Indirectly related	A transcriptional profile of aging in the human kidney.	Rodwell GE et al.	PLoS Biol. 2004	PMID:15562319
Indirectly related	Functional gene expression analysis of clonal plasma cells identifies a unique molecular profile for light chain amyloidosis.	Abraham RS et al.	Blood. 2005	PMID:15388584
Indirectly related	Microarray analysis of differentiation-specific gene expression during 3T3-L1 adipogenesis.	Burton GR et al.	Gene. 2004	PMID:15033539
Indirectly related	Identification of the apoptosis activation cascade induced in mammary carcinomas by energy restriction.	Thompson HJ et al.	Cancer Res. 2004	PMID:14973070
Indirectly related	Stemness": transcriptional profiling of embryonic and adult stem cells."	Ramalho-Santos M et al.	Science. 2002	PMID:12228720
Indirectly related	Systematic screen for human disease genes in yeast.	Steinmetz LM et al.	Nat Genet. 2002	PMID:12134146
Indirectly related	Influences of aging and caloric restriction on the transcriptional profile of skeletal muscle from rhesus monkeys.	Kayo T et al.	Proc Natl Acad Sci U S A. 2001	PMID:11309484
Liver	Genomic and proteomic analysis reveals a threshold level of MYC required for tumor maintenance.	Shachaf CM et al.	Cancer Res. 2008	PMID:18593912
Liver	Epigenetics of gene expression in human hepatoma cells: expression profiling the response to inhibition of DNA methylation and histone deacetylation.	Dannenberg LO et al.	BMC Genomics. 2006	PMID:16854234
Liver	Integrating time-course microarray gene expression profiles with cytotoxicity for identification of biomarkers in primary rat hepatocytes exposed to cadmium.	Tan Y et al.	Bioinformatics. 2006	PMID:16249259
Unrelated cancer	Integrated analysis of DNA methylation and gene expression reveals specific signaling pathways associated with platinum resistance in ovarian cancer.	Li M et al.	BMC Med Genomics. 2009	PMID:19505326
Unrelated cancer	Genetic variants in apoptosis and immunoregulation-related genes are associated with risk of chronic lymphocytic leukemia.	Enjuanes A et al.	Cancer Res. 2008	PMID:19074885
Unrelated cancer	Evolutionary origins of human apoptosis and genome-stability gene networks.	Castro MA et al.	Nucleic Acids Res. 2008	PMID:18832373
Unrelated cancer	Ganoderma lucidum polysaccharides in human monocytic leukemia cells: from gene expression to network construction.	Cheng KC et al.	BMC Genomics. 2007	PMID:17996095
Unrelated cancer	Comparative analysis of genes regulated in acute myelomonocytic leukemia with and without inv(16)(p13q22) using microarray techniques, real-time PCR, immunohistochemistry, and flow cytometry immunophenotyping.	Sun X et al.	Mod Pathol. 2007	PMID:17571080
Unrelated cancer	Analysis of gene expression identifies PLAB as a mediator of the apoptotic activity of fenretinide in human ovarian cancer cells.	Appierto V et al.	Oncogene. 2007	PMID:17213814
Unrelated cancer	The expression of FHIT, PCNA and EGFR in benign and malignant breast lesions.	Terry G et al.	Br J Cancer. 2007	PMID:17164758
Unrelated cancer	Identification of differentially expressed genes in cutaneous squamous cell carcinoma by microarray expression profiling.	Nindl I et al.	Mol Cancer. 2006	PMID:16893473
Unrelated non-cancer	Progress towards in vivo use of siRNAs.	Behlke MA. et al.	Mol Ther. 2006	PMID:16481219
Unrelated non-cancer	Global gene expression in human myocardium-oligonucleotide microarray analysis of regional diversity and transcriptional regulation in heart failure.	Kaab S et al.	J Mol Med (Berl). 2004	PMID:15103417
